# Physiologic maturation is both extrinsically and intrinsically regulated in progenitor-derived neurons

**DOI:** 10.1038/s41598-020-58120-5

**Published:** 2020-02-11

**Authors:** Praseeda Venugopalan, Evan G. Cameron, Xiong Zhang, Michael Nahmou, Kenneth J. Muller, Jeffrey L. Goldberg

**Affiliations:** 1 Neuroscience Program, University of Miami, Miami, FL 33136 USA; 2 Shiley Eye Institute, University of California, San Diego, CA 92093 USA; 3 Department of Physiology & Biophysics, University of Miami Miller School of Medicine, Miami, FL 33136 USA; 4 Byers Eye Institute, Stanford University, Stanford, CA 94303 USA

**Keywords:** Neuronal development, Ion channels in the nervous system

## Abstract

During development, newly-differentiated neurons undergo several morphological and physiological changes to become functional, mature neurons. Physiologic maturation of neuronal cells derived from isolated stem or progenitor cells may provide insight into maturation *in vivo* but is not well studied. As a step towards understanding how neuronal maturation is regulated, we studied the developmental switch of response to the neurotransmitter GABA, from excitatory depolarization to inhibitory hyperpolarization. We compared acutely isolated retinal ganglion cells (RGCs) at various developmental stages and RGCs differentiated *in vitro* from embryonic retinal progenitors for the effects of aging and, independently, of retinal environment age on their GABA_A_ receptor (GABA_A_R) responses, elicited by muscimol. We found that neurons generated *in vitro* from progenitors exhibited depolarizing, immature GABA responses, like those of early postnatal RGCs. As progenitor-derived neurons aged from 1 to 3 weeks, their GABA responses matured. Interestingly, signals secreted by the early postnatal retina suppressed acquisition of mature GABA responses. This suppression was not associated with changes in expression of GABA_A_R or of the chloride co-transporter KCC2, but rather with inhibition of KCC2 dimerization in differentiating neurons. Taken together, these data indicate GABA response maturation depends on release of inhibition by developmentally regulated diffusible signals from the retina.

## Introduction

In the central nervous system (CNS), neurons are born from stem or progenitor cells, and as the brain develops, the newborn neurons acquire physiological properties that play a major role in their survival and in the development of networks of highly specific synaptic connections^1–3^. Several developmental milestones, including expression of excitable channels^4,5^ and maturation of the actions of neurotransmitters^6–8^, reflect a shift in function during development and are required before a neuron can operate as a functional unit within a mature neuronal circuit. Reaching such developmental milestones distinguishes a physiologically maturing neuron.

Are either stem cells or immature neurons extrinsically signaled to mature physiologically, or are such developmental changes intrinsically programmed in a cell-autonomous fashion? The retina is a favorable part of the CNS to address these questions, as retinal progenitor cells and their progeny can be differentiated and studied in purified or enriched cultures^9,10^, permitting study of neuronal development under controlled conditions. When retinal and other neurons first arise from neural progenitor cells *in vivo*, they differ from mature neurons in several critical ways. For example, newly-formed retinal ganglion cells (RGCs) have greater intrinsic capacity for rapid axon growth, which transitions towards dendrite growth ability early in development^11^. The composition of NMDA receptor subunits also changes as RGCs mature, playing an important role in RGC-bipolar cell synaptogenesis and circuit refinement^12,13^. A particularly dramatic developmental landmark is the reversal of responses to the neurotransmitter GABA from excitatory early in development, helping to drive activity, to inhibitory in mature neurons^14^. This switch is achieved not through modifications of the GABA receptor itself, but through an increase in the chloride gradient produced primarily by action of the chloride co-transporter KCC2, so that chloride moves *in* rather than *out* through activated GABA_A_ receptors^14–16^. Some studies have indicated that diffusible factors from the superior colliculus^17^ or GABA itself^18^ may contribute to this developmental reversal in GABA response. These examples demonstrate the complex developmental changes neurons undergo on their path to maturity and formation of functional neural circuits.

In contrast to normal development *in vivo*, very little is known about the developmental maturation of neurons generated from stem cells or progenitor cells *in vitro*. This has become particularly important given the current interest in investigating stem cells both for disease modeling and cell transplantation therapy. The present experiments were designed to study neurons generated from embryonic retinal progenitors *in vitro*, particularly their maturation, using the response to GABA receptor activation as one such indicator. These data focus on the cellular and molecular mechanisms associated with the shift from excitation to inhibition, and advance a new model in which intrinsically regulated maturation of GABA_A_ responses in RGC-like neurons is inhibited by diffusible molecules released by the immature retina that work to regulate KCC2 activity by inhibiting dimerization.

## Results

### GABA_A_ receptor activation is excitatory in embryonic progenitor-derived RGCs

**Figure 1:**
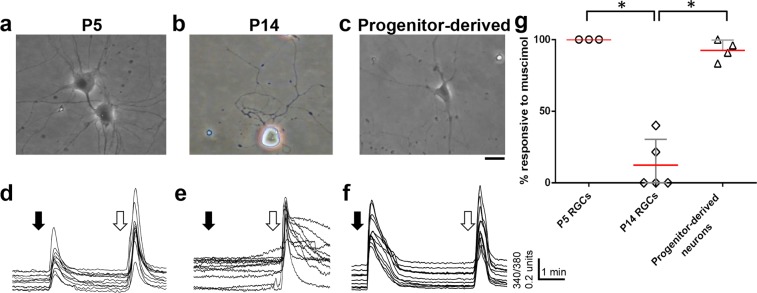
Progenitor-derived neurons cultured for 7 days *in vitro* (DIV) exhibited immature responses to GABA_A_ receptor activation. (**a**–**c**) Representative pictures of purified P5 RGCs, purified P14 RGCs, and progenitor-derived neurons, as marked. Scale bar is 25 µm. (**d**–**f**) Cells in (**a**–**c**) were loaded with the calcium-responsive fluorophore fura-2-AM and stimulated with pulses of muscimol (black arrow) or KCl (white arrow); representative calcium fluorescence traces from each of the three cell cultures are shown. (**g**) Summary statistics of muscimol responses show that progenitor-derived neurons cultured for 7 DIV responded like P5 RGCs, with increased intracellular calcium, consistent with the immature GABA_A_ receptor response, and significantly different from the responses of the P14 RGCs. In this and other figures, each point corresponds to the fraction of cells that responded to muscimol. Red lines are means; error bars are S.D.s. *p < 0.001.

Muscimol is a selective, potent activator of GABA_A_ receptors in neurons^19^ and was used to assess the GABA_A_ response in progenitor-derived neurons using calcium imaging with fura 2-AM, since Ca^2+^ levels rise when the neurons depolarize^20^. First, as a control using cultured RGCs to confirm previous results^17^, muscimol application elicited a strong elevation of intracellular calcium in purified, cultured RGCs from early postnatal (postnatal day 5, P5) rats (Fig. 1a,d), indicative of an immature neuronal state. As expected, no calcium elevation was observed in RGCs purified by postnatal day 14 (P14) (Fig. 1b,e), although all neurons responded to a depolarizing KCl-stimulus used as a positive control throughout these experiments (open arrows in Fig. 1d–f). Statistical analysis confirmed that significantly fewer RGCs responded to muscimol by postnatal day 14 (Fig. 1g, p = 0.0004), consistent with a switch in GABA_A_ responsiveness *in vitro* between P5-P14, similar to the time course *in vivo*.

Do progenitor-derived neurons generated *in vitro* exhibit a similar reversal in GABA responses? As a step towards understanding the maturation of GABA_A_ responses in newly formed neurons, we differentiated RGC-like neurons *in vitro* from cultures of embryonic day 14 (E14) retinal progenitor cells in serum-free medium containing BDNF (50 ng/ml) and CNTF (10 ng/ml), as described in Methods. By 7 days in culture (7 DIV), chosen as a time point corresponding to an early postnatal age *in vivo*, cells with neuronal morphology responded to muscimol by elevating intracellular calcium, reflecting a depolarization (Fig. 1c,f). Muscimol application elevated intracellular calcium in over 90% of the cells, indicative of an excitatory response similar to responses seen in early postnatal neurons (Fig. 1g). Calcium levels in cells with non-neuronal, glia-like morphology within the same fields of view did not change in response to muscimol or KCl. These data show that newly formed neurons from embryonic retinal progenitors predominantly exhibit an immature response to GABA_A_ receptor activation.

**Figure 2:**
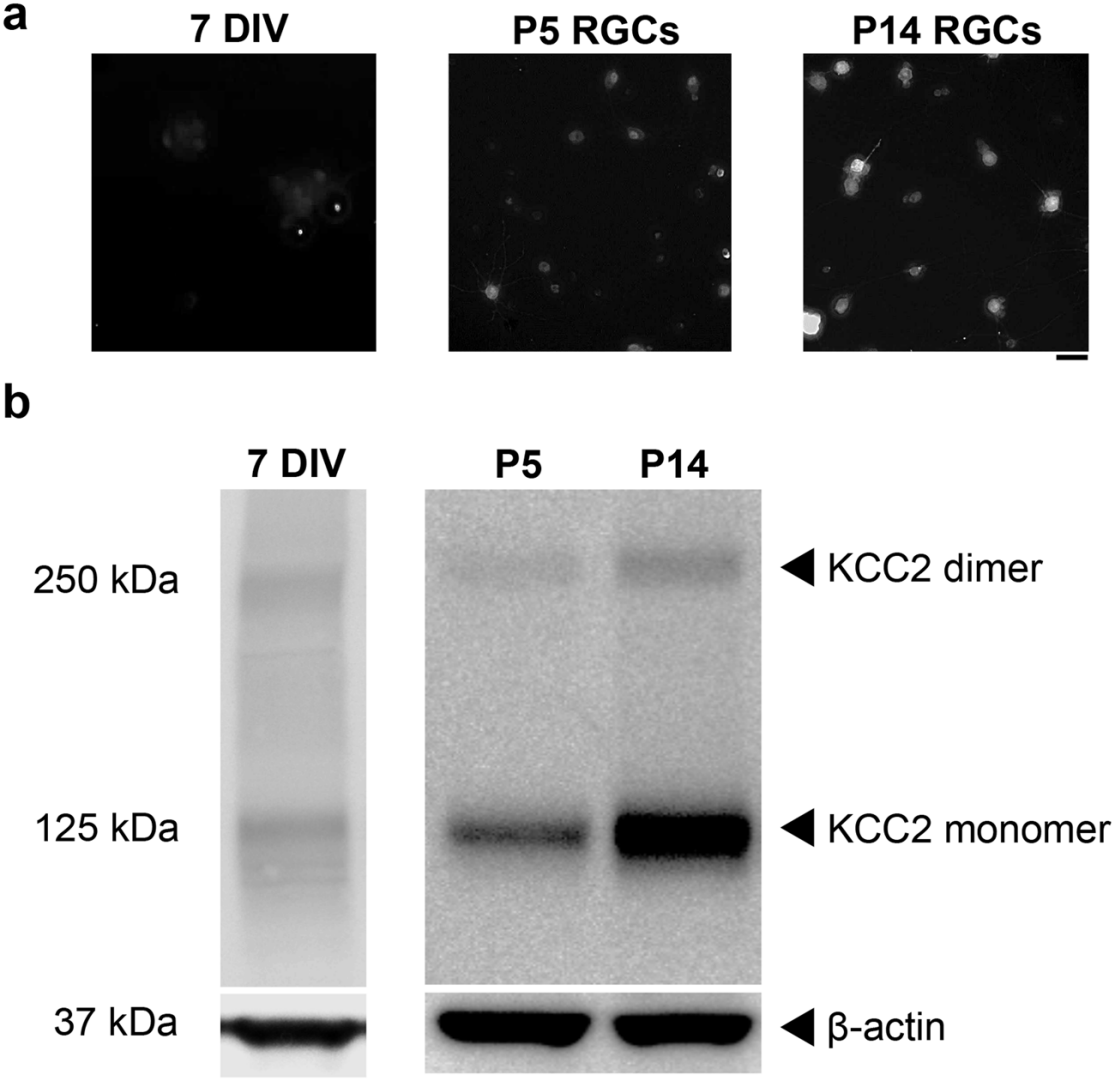
KCC2 expression is low in progenitor-derived neurons by 7 DIV. (**a**) Little KCC2 immunofluorescence was observed in progenitor-derived neurons and P5 RGCs compared with P14 RGCs. Scale in (**a**) is 50 µm. (**b**) Faint protein bands corresponding to KCC2 monomer and dimer were observed in progenitor derived neurons (7 DIV) and early postnatal retinas (P5) while retinal KCC2 was greatly upregulated by postnatal day 14 (P14). 7 DIV and P5/P14 blots were run separately.

Reversal of GABA responses during development has been shown to correlate with increased expression of the KCl co-transporter KCC2^15^. Fluorescence immunocytochemistry showed little to no KCC2 immunofluorescence in progenitor-derived neurons (Fig. 2a). This was confirmed by western blotting for KCC2 in protein samples from progenitor-derived cultures after 7 DIV (Fig. 2b). The low expression of KCC2 in progenitor-derived neurons after 7 DIV was no more than the low expression levels detected in early postnatal purified RGCs (Fig. 2b). Thus, both molecular and cellular data related to GABA_A_ receptor response properties are consistent with an immature state of physiological maturation for progenitor-derived neurons generated *in vitro*.

### GABA_A_ response matures with extended time and is inhibited by early postnatal retinal cells

**Figure 3:**
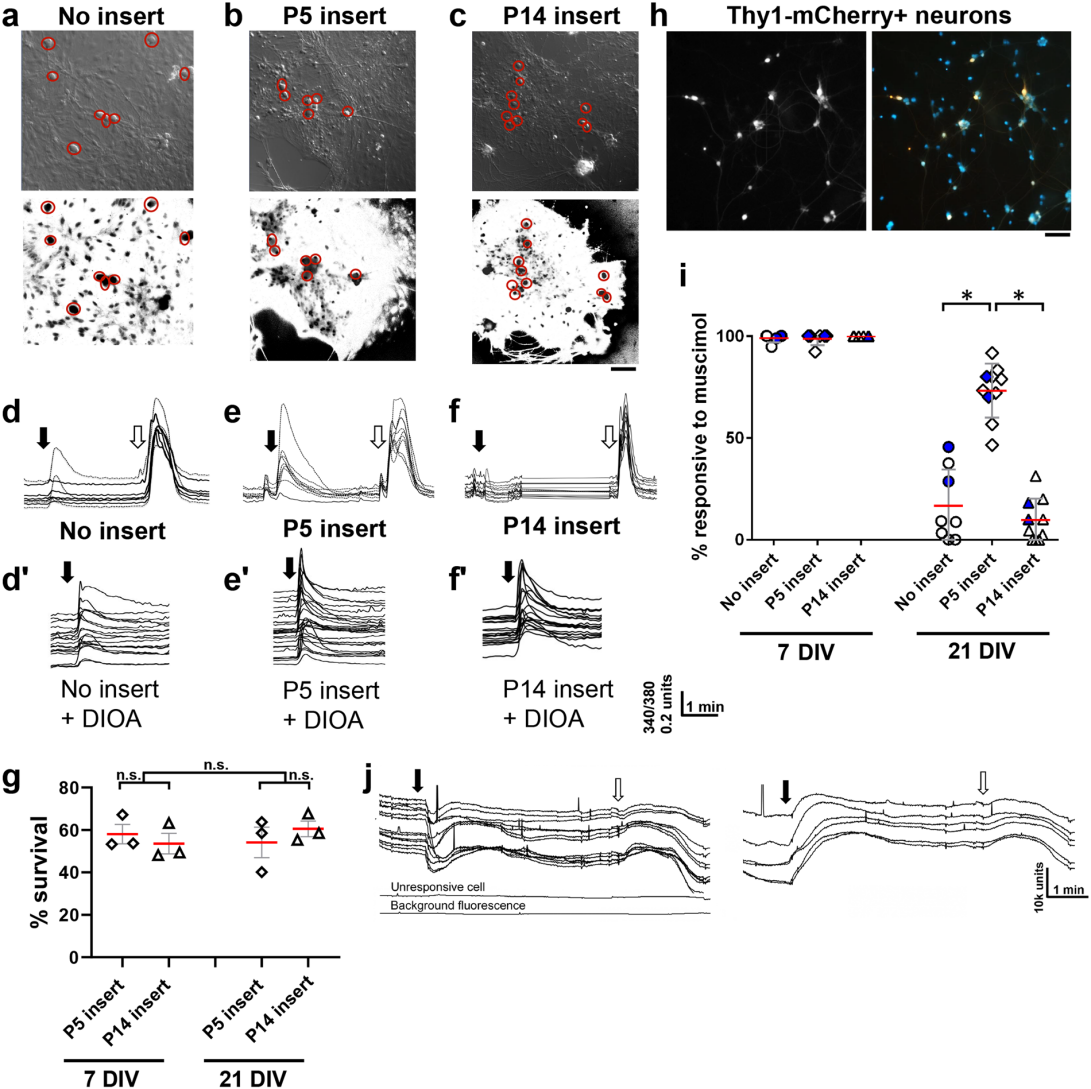
Early postnatal retinal cells inhibit maturation of GABA_A_ responses. (**a**–**c**) Representative images of progenitor-derived neuron cultures at 21 DIV (DIC images above) and the regions of interest isolating cells with neuronal morphology (red circles, calcium dye images below), in 3 insert conditions as marked. Scale bar is 50 µm. (**d**–**f**) Representative calcium traces as in Fig. 1, showing calcium responses to muscimol under the indicated conditions, with consistently large responses with the P5 insert. (**d**’–**f**’) Parallel cultures treated with the KCC2 blocker DIOA restored calcium responses in matured progenitor-derived cultures. (**g**) P5 and P14 retinal cell survival in inserts was similar at 7 DIV and 21 DIV. P5 and P14 retinas were dissociated, cultured in inserts for 7 DIV and 21 DIV, and then examined and counted as described in Methods. Differences in cell numbers and survival were not significant (n.s). (**h**) (left panel) mCherry^+^ progenitor-derived neurons and presumptive RGCs; (right panel) fura 2-AM loaded progenitor-derived neurons. Scale bar is 50 µm. (**i**) Summary statistics demonstrating that by 21 DIV, the majority of progenitor-derived neurons cultured without inserts no longer responded to muscimol application. This maturation was suppressed in the presence of P5 retinal suspension inserts, but not in the presence of P14 retinal suspension inserts. Calcium responses in Thy1-mCherry-expressing neurons are highlighted by blue filled symbols. Red lines are means; error bars are S.D.s. *p < 0.05. (**j**) In FLIPR voltage-sensitive dye imaging of progenitor-derived cultures treated with muscimol after 14 DIV, approximately 60% of cells exhibited transient decrease in fluorescence, indicative of hyperpolarization (left panel), while the remaining 40% of cells showed elevated fluorescence, indicative of depolarization (right panel). All progenitor-derived cells with neuronal morphology exhibited elevated fluorescence in response to KCl; one example unresponsive cell (as marked) did not have neuronal morphology.

Previous findings have suggested that reversal of GABA responses in RGCs may not be intrinsically regulated but rather requires an extrinsic, or non-cell autonomous, signal^17^. To test whether the retinal environment at different developmental stages could influence maturation of GABA_A_ response properties, embryonic retinal progenitors were cultured alone (“no insert”) or in the presence of a P5 or P14 retinal suspensions in hanging cell inserts (“P5 insert” and “P14 insert,” respectively). The progenitor derived neurons cultured under the inserts were imaged in similar fashion (Fig. 3a–c) and their calcium responses (Fig. 3d–f) or responses in the presence of KCC2 inhibitor (DIOA, Fig. 3d’–f’) recorded. We found that after short-term culture (7 DIV), all progenitor-derived neurons exhibited immature responses to GABA_A_ receptor activation irrespective of culture conditions (Fig. 3i). However, by 21 days in culture (21 DIV), significantly fewer neurons in the “no insert” condition responded to muscimol application with calcium elevation (no insert, p = 0.019), indicating that they were not depolarized by GABA_A_ receptor activation (Fig. 3d, 3i). Thus, the default pathway with sufficient time in a mixed differentiating culture of retinal progenitor cells in the absence of soluble signals from other postnatal retinal cells is towards physiological maturation.

Strikingly, neurons cultured 21 DIV with P5 inserts depolarized in response to muscimol, indicating that the immature, depolarizing response persisted in the presence of P5 retinal cells (Fig. 3e,i). This was significantly different from the lack of response seen in the absence of insert and those co-cultured with P14 retinal suspensions (Fig. 3d,f,i) (p < 0.001). Importantly, there were no significant differences in survival or total cell numbers of the P5 or P14 inserts themselves (Fig. 3g). A similar trend in calcium responses to muscimol was also observed in mCherry^+^ neurons generated from retinal progenitors expressing Thy1-promoted mCherry (Fig. 3h,i). Since in the rodent retina most Thy1-expressing cells are RGCs, these data support the conclusion that the cells in this study had differentiated into RGC-like cells.

Treating parallel progenitor-derived cultures with the KCC2 blocker DIOA restored calcium responses upon GABA_A_ receptor activation in the matured cultures, demonstrating that the lack of response was not an artefact caused by a dysfunctional receptor (Fig. 3d’–f’).

Voltage sensitive dye imaging was used to further verify that the absence of a response to muscimol was indicative of a mature, hyperpolarizing response. In progenitor-derived cultures grown for 14 days, neurons loaded with the FLIPR dye exhibited fluorescence elevation (depolarization) as well as decreased fluorescence (hyperpolarization) in response to muscimol application (Fig. 3j). The consistent depolarization in response to KCl was sometimes later followed by an apparent hyperpolarization, characteristic of the FLIPR voltage-sensitive dye recording. Overall, these voltage dye and calcium imaging-based data together support a model in which the early postnatal retinal environment strongly inhibits acquisition of mature GABA_A_ response properties in neurons, including RGCs, generated from retinal progenitors.

### Inhibition of maturation by early postnatal retinal environment is associated with suppression of KCC2 dimerization

**Figure 4:**
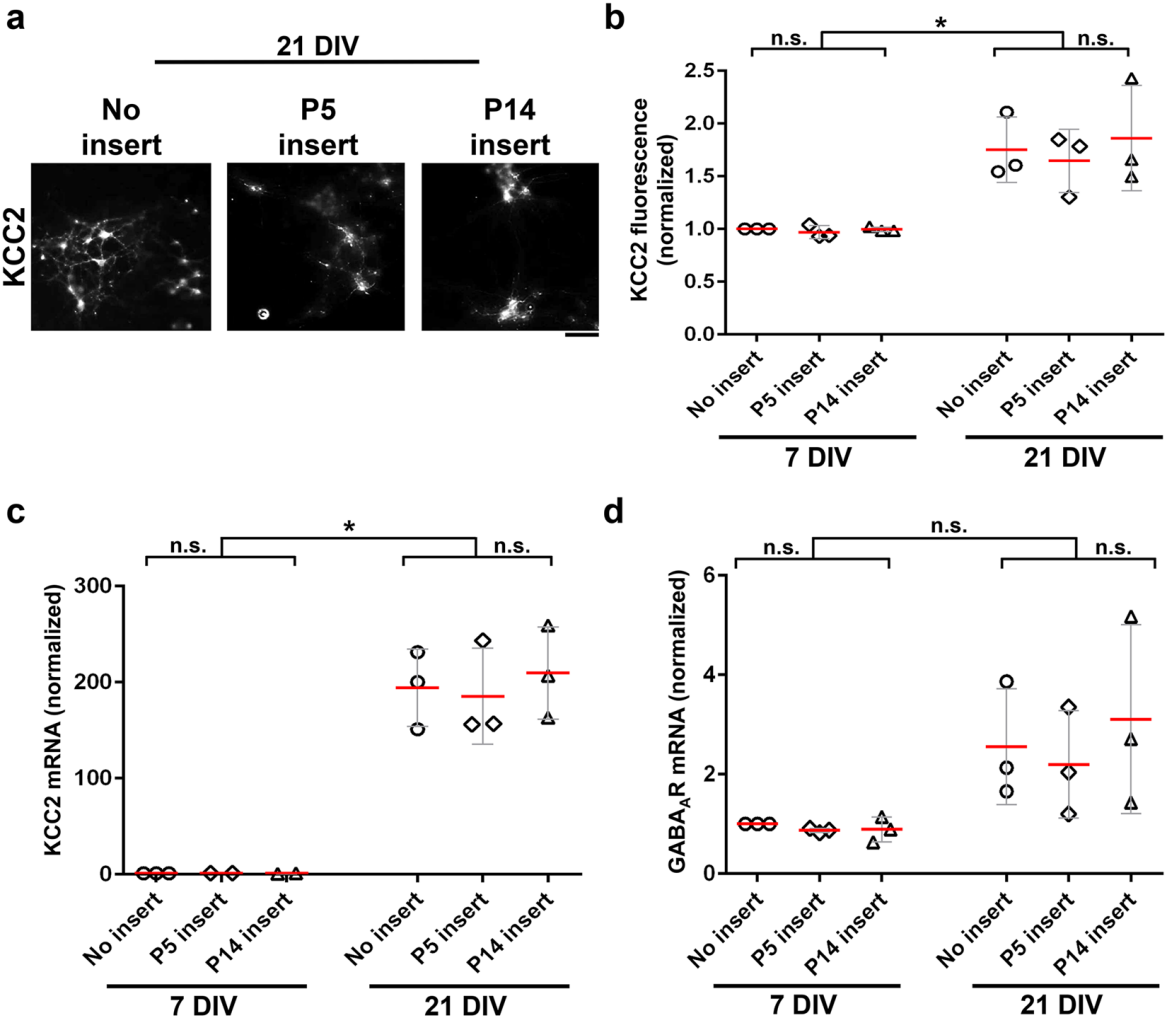
KCC2 is upregulated in progenitor-derived neurons by 21 DIV. **(a**) Example images of KCC2 immunofluorescence in retinal progenitor-derived neurons at 21 DIV in all three insert conditions as marked. Scale bar is 50 µm. (**b**) Immunofluorescence measurements of relative KCC2 protein levels in all 3 conditions at 7 DIV and 21 DIV. (**c**) qPCR data showed >150-fold increase in KCC2 mRNA expression in progenitor-derived neurons from 7 DIV to 21 DIV in all three insert conditions, but no difference among the three insert conditions. (**d**) qPCR data show no significant differences in GABA_A_ receptor subunit expression between 7 DIV and 21 DIV or among the three insert conditions. In (**b**–**d**) red lines indicate means; error bars, S.D.s; n.s. difference not significant; *p < 0.05. Immunofluorescence and qPCR data were normalized to values at 7 DIV with no insert.

To determine whether the functional effect of the insert cultures could be explained by changes in expression of KCC2, we immunostained progenitor cells for KCC2 after they had been cultured under the three different insert conditions: no insert, P5 insert and P14 insert. Accordingly, we found a significant increase in KCC2 immunofluorescence across all three conditions with longer time in culture (Fig. 4a,b; no insert, p = 0.0136; P5 insert, p = 0.0184; P14 insert, p = 0.0398). This was confirmed by qPCR data, which showed an approximately 200-fold increase in KCC2 mRNA expression after 21 DIV (Fig. 4c; no insert, p = 0.0143; P5 insert, p = 0.0238; P14 insert, p = 0.0172). Thus, the suppression of GABA_A_ receptor response maturation seen in the presence of P5 retinal cells did not appear to be mediated through inhibition of KCC2 expression. We also tested the possibility that downregulation of GABA_A_ receptor in control and P14 retinal cell insert samples caused the loss of GABA response, but qPCR for GABA_A_ receptor subunit alpha 1 showed no significant difference in mRNA expression between conditions, suggesting that the functional differences in responses to muscimol were not due to loss of GABA_A_ receptor expression (Fig. 4d).

**Figure 5:**
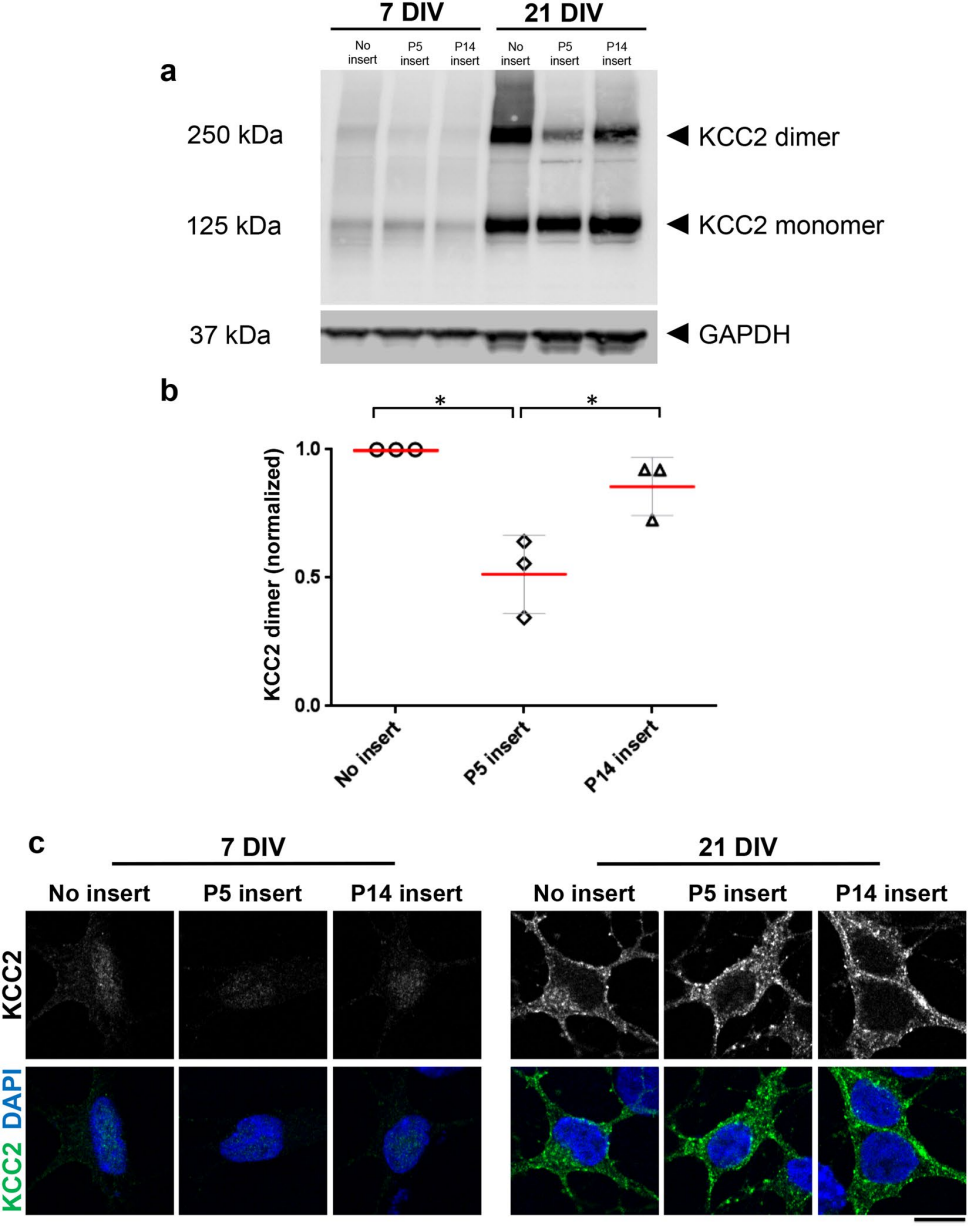
Inhibition of maturation by early postnatal retinal cells is associated with suppression of KCC2 dimerization. **(a**) Western blot under non-reducing conditions detected KCC2 monomer and dimer at expected molecular weights. KCC2 monomers and dimers were upregulated in progenitor-derived neurons from 7 DIV to 21 DIV, but with less dimer in neurons cultured in the presence of P5 retinal suspension inserts. (**b**) Density measurements normalized to values from 21 DIV, no insert, confirmed that there was significantly less dimer in the presence of P5 retinal suspension inserts. (**c**) High magnification of KCC2 immunofluorescence in 7 DIV and 21 DIV retinal progenitor-derived neurons with inserts as marked. KCC2 is upregulated and externalized to the plasma membrane after 21 DIV in all co-culture conditions. Scale bar is 10 µm. In (**b**) red lines show means; error bars, S.D.s; *p < 0.05.

Previous studies have implicated post-translational modifications such as phosphorylation^21,22^ and oligomerization^23^ as affecting KCC2 activity. To test whether the excitatory response to muscimol seen in 21 DIV progenitor-derived neurons co-cultured with P5 inserts might have been due to such a post-translational change in KCC2, we used western blotting to examine KCC2 dimers and oligomers. Consistent with the immunofluorescence and qRT-PCR data in Fig. 4a–c, little KCC2 monomer or dimer was detected in 7 DIV progenitor-derived neurons (Fig. 5a). Conversely, by 21 DIV there was a considerable upregulation of KCC2 monomer and dimer in all three culture conditions; however, significantly less KCC2 dimer was detected in 21 DIV neurons cultured with P5 inserts (Fig. 5a,b; p = 0.03). It has been well established that KCC2 is externalized to the plasma membrane of mature neurons, where it oligomerizes to form functional KCC2 dimers^21–23^. Accordingly, we asked whether KCC2 distribution on the plasma membrane was affected by the early retinal environment using fine immunocytochemistry. While little KCC2 was detected in 7 DIV progenitor-derived neurons, we found KCC2 was highly enriched in discrete puncta on the plasma membrane of 21 DIV neurons across all conditions (Fig. 5c). Taken together with our expression and biochemical data, these results indicate that the inhibitory effect of the early postnatal retinal cells on maturation of GABA responses is associated with less dimerization of KCC2 at the plasma membrane, not with expression nor translocation, supporting the hypothesis that factors secreted from the early retina inhibit the GABA switch by suppressing dimerization-dependent KCC2 activity.

## Discussion

Here we define a new inhibitory mechanism for the physiologic maturation of a CNS neuron. Neurons undergo several developmental changes morphologically and functionally prior to the formation of mature neural circuits^24^. These changes are tightly regulated, presumably to assure precise formation and fine-tuning of networks. The excitatory role of GABA in early development is essential to several processes in neural development including proliferation, migration and neurite growth^25–27^ as well as synapse formation^28–30^. Studies have also suggested that this excitatory action of GABA is important for synaptic integration by newly-formed neurons in the adult dentate gyrus and olfactory bulb^31,32^. Thus, the excitatory action of GABA has a strong influence on the development and refinement of the nervous system.

We observed that after a short time in culture, progenitor-derived neurons exhibit immature GABA_A_ responses. This likely reflects the natural progression of physiologic development *in vivo*, as progenitor-derived neurons eventually developed mature GABA_A_ responses with significantly longer time in culture. A previous study had not replicated this progression for purified RGCs in culture^17^, as purified RGCs may require extrinsic signals that we included in the molecules used to drive differentiation of RGCs from progenitors. Other signals may be missing in pure cultures of RGCs but present in progenitor-derived cultures that generate not just neurons, but also other, non-neuronal glia-like cells; this could include a signal like neuroligin-2 (NL2), which has been shown to be a potential regulator of KCC2 expression and GABA response reversal in cortical neurons^33^. Such mixed cultures could provide the requisite signals for the newly-formed neurons to mature with time and suggest a useful model system for future experiments targeted at identifying such signals.

The maturation of the GABA response *in vitro* led to an additionally unexpected finding that soluble factors from P5 and not P14 retinal suspensions inhibited the maturation of that response. This difference was apparently not due to differences in the cell viability of P5 vs P14 inserts, as both preparations exhibited similar survival across all conditions. Again, using this model system, future experiments could determine the identity and properties of the inhibitory signal or signals, the specific cells that produced them, and whether bidirectional signaling is required between the P5 retinal cells and the nascent RGCs. Nevertheless, the discovery of an intrinsic developmental switch in GABA receptor physiology for which there is an extrinsic inhibition is novel.

Although we first expected KCC2 expression to dictate this maturation timeline, KCC2 expression was upregulated under all culture conditions with sufficient time, including those inhibiting maturation of GABA_A_ receptor responses. However, a difference was observed in post-translational modification of KCC2, specifically in dimerization, which is known to influence function^23,34^. This supports the hypothesis that the early retinal environment inhibits GABA_A_ response maturation by suppressing formation of functional, dimerized KCC2 co-transporter. Phosphorylation of a key tyrosine residue has previously been shown to be required for KCC2 oligomerization^34^, so molecules secreted by cells in the early postnatal retinal suspension may disrupt this as yet unidentified signaling pathway. It remains to be determined if these factors mediate a delay in the acquisition of mature responses or can completely inhibit the process, perhaps over longer times in culture. Indeed, maturation of GABA_A_ receptor activity might depend on a balance of molecules that inhibit and facilitate maturation, and it would be interesting to know whether identified signals can induce a reversal of maturation in developmentally mature neurons *in vitro* or *in vivo*.

Our results point to changes in KCC2 underlying the maturation of the response to GABA, but they do not eliminate the possibility that other mechanisms might also participate. For example, there could be a contribution of the NKCC1 transporter, which rises in non-neuronal cells and in neurons, and initially accounts for the lowered Nernst potential for chloride. Another formal possibility would be an action of background synaptic interactions, such that muscimol produces disinhibition, but the uniform results with calcium and voltage-sensitive dye imaging across neurons in preparations showing delayed maturation argue against it.

As a technical matter, calcium imaging is an indirect but well-established measure of the voltage response, it permits rapid screening of large numbers of cells, and it is useful for the detection of depolarization whether or not some of the measured calcium is released from intracellular stores. Moreover, the results with voltage indicator dye were consistent with those from calcium imaging, but in future studies it would be of interest to add pharmacological approaches, such as application of thapsigargin to deplete calcium stores in endoplasmic reticulum or 2-Aminoethoxydiphenyl borate (2-APB) to block IP3-mediated calcium release, to determine if some of the calcium rise is from such intracellular release. Direct electrophysiological recordings would also complement these data, but our attempts at recording from the small, immature cells in these cultures proved difficult and unreliable.

The fact that cultured neurons, which changed their responses to muscimol, did not exhibit calcium responses in baseline readings, supports the notion that if background synaptic activity were present, it did not provide measurable excitation of one cell by another. This is of interest in part to address the theoretical question of whether cellular heterogeneity and synaptic interactions might contribute to the observed changes in physiology. Not only did all immature neurons depolarize at early stages, but with maturation the transition to hyperpolarization measured with voltage sensitive dyes was consistent with only a subset of cells retaining calcium signals.

Stem cell-derived neurons are an often-studied source of donor material for cell replacement, and given the importance of GABA during neuronal development, it might be preferable to use cells with specified GABA response properties, as well as other indicators of physiologic maturation. Our study indicates that the excitatory response to GABA is present transiently in newly-born neurons generated *in vitro* from embryonic retinal progenitors. This may guide a choice between ‘immature’ and ‘mature’ neurons for effective cell replacement following *in vivo* transplantation. Based on available literature on adult born neurons *in vivo*^31^, neurons that are depolarized by GABA may be a more viable donor population when synaptic integration is desired. Thus, identification of molecules that inhibit acquisition of mature, inhibitory GABA responses might foster generation of immature stem cell or progenitor cell-derived neurons that are better able to make suitable synaptic connections with host neurons.

Taken together, our data shed light on the mechanism behind functional maturation of neurons from progenitor cells. We anticipate that understanding the regulation of developmental changes in neurons and being able to influence them under controlled conditions will be critical to generating better disease models and cell replacement strategies.

## Materials and Methods

### Cell purification and culture

RGCs from early and late postnatal rats were purified and cultured as published^35^. Briefly, whole retinas were extracted and dissociated with papain, followed by sequential immunopanning with the Thy1 antibody to yield 99.5% pure RGCs. Purified RGCs were cultured for 3–4 days before calcium imaging. Embryonic retinal progenitors were obtained from E14 rat embryos by papain (Worthington; LS003124) digestion of dissected retinas. Approximately 100,000 purified RGCs or retinal progenitors were plated on individual poly-D-lysine- and laminin-coated coverslips in 24 well plates in serum-free RGC growth medium containing sodium pyruvate, N-acetyl cysteine, L-glutamine, Sato supplement, insulin, BDNF (brain-derived neurotrophic factor), CNTF (ciliary neurotrophic factor), and forskolin, as described previously^36^, and neural supplement GS21 (MTI-GlobalStem) was added. The cells were cultured for 7–21 days with media changes every 3 days. After 4–5 days *in vitro*, RGC-like neurons were generated from the retinal progenitor cells along with some glia-like cells.

Hanging cell culture inserts (0.4 µm pore size, Millipore) were prepared using papain-digested retinas from P5 and P14 rats; these were placed within the culture wells hanging over the retinal progenitors. The contents of the hanging inserts were left undisturbed for the duration of culture, while the media were replenished through media changes in the main chamber. Survival of P5 and P14 retinal cell suspensions was determined by dye exclusion. Briefly, cells were detached from the hanging culture inserts by moderate pipetting, diluted 1:1 with Trypan blue and counted using a hemocytometer.

### Calcium imaging and analysis

Cover slips with cultured cells were incubated for 30 min at room temperature with 5 µM fura 2-AM (Life Technologies; F-1221) in Tyrode’s solution: (in mM) NaCl 129, KCl 5, CaCl_2_ 2, MgCl_2_ 1, glucose 30, and HEPES 25, pH 7.4, containing 1 mg/ml bovine serum albumin. The cells were washed with Tyrode’s solution and placed in the imaging chamber (RC-26GLP, Warner Instruments). Calcium imaging was performed on an inverted fluorescence microscope (AxioObserver, Zeiss) with a fast switching light source (DG-4, Sutter Instruments) using MetaFluor software (Molecular Devices) working within a linear range of the camera. Regions of interest (ROIs) were drawn around brightly labeled cell bodies corresponding to cells with neuronal morphology observed under DIC illumination. As a control, in each field of view 1–2 ROIs were also drawn around cells with non-neuronal, glia-like morphology. Calcium responses within these regions of interest were observed as changes in fluorescence ratio (340/380) upon sequential stimulation with muscimol (100 µM, Worthington) and KCl (30 mM) in Tyrode’s solution. The muscimol dose was within the reported linear range of dose response curves. Test solutions were replaced with Tyrode’s solution following each stimulus. A response was considered positive if the change in fluorescence ratio exceeded six times the standard deviation in baseline values. With this criterion, cells were scored as responsive or non-responsive to a specific stimulus. More than 25 cells were tested and analyzed in each experiment. Each point on the summary plots corresponds to the fraction of cells in each experiment that responded to muscimol. 100% of the neuronal cells responded to KCl. As controls, 1–2 cells of non-neuronal morphology were also measured; these cells did not respond to KCl.

Where indicated, to determine that a lack of calcium response was because the chloride current had reversed, the KCC2 inhibitor dihydroindenyl-oxy-alkanoic acid (DIOA, Sigma; D129) was added and the response to muscimol retested. DIOA was dissolved in 0.1 N NaOH (20 mM stock) and was added at a final concentration of 20 µM in Tyrode’s solution. The appearance of a response in DIOA was used to confirm that the GABA_A_ receptor remained active and the Nernst potential for chloride had shifted.

### Immunostaining and analysis

Following calcium imaging experiments, coverslips were fixed with 4% paraformaldehyde in 1X PBS. The cells were blocked and permeabilized (5% goat serum; 0.2% Triton X-100) before staining for co-transporter KCC2 (1:200 rabbit polyclonal Ab49917, Abcam) overnight at 4 °C. Fluorophore-conjugated anti-rabbit secondary antibodies were used to label the primary antibodies and unlabeled controls. Stained samples were imaged on a Zeiss Axio Observer 7 using the same imaging parameters between test groups. KCC2 fluorescence intensity was obtained from non-saturated immunostained images by measuring the mean gray values of over 100 cells per condition per experiment using Fiji^37^. High magnification KCC2 images were acquired on a Zeiss 880 confocal microscope at 63X using Airyscan Fast.

### Voltage sensitive dye imaging

Progenitor cells were cultured in 24-well plates on coverslips for 14 days. Coverslips were moved to the imaging chamber and washed with Tyrode’s solution. Red FLIPR dye (FLIPR Membrane Potential Assay Kit; Molecular Devices) was reconstituted to a final concentration of 20 µM and added to the imaging chamber. The cells were incubated for 30 mins at 37 °C. Muscimol (100 µM) and KCl (30 mM) were added to the FLIPR dye solution and applied to the cells using gravity perfusion while the fluorescence responses were imaged by taking images every 500 ms. Fluorescence changes were analyzed using Fiji software and values plotted in Excel.

### Western blotting and analysis

Progenitor cells with and without hanging inserts were cultured in 6-well plates for 7 or 21 days. The cultures were harvested by mechanical dissociation in ice-cold 1X protease inhibitor (100X, ThermoFisher; 78440) in PBS and centrifuged at 16,000 rpm at 4 °C to lyse the cells. The membrane pellet was frozen and processed for protein extraction by treating with 1% DDM (ThermoFisher; BN2005) and 1X protease inhibitor in PBS for 1.5 hours on ice. The samples were centrifuged at 16,000 rpm at 4 °C to remove non-solubilized fragments, and the protein concentration in the supernatant was measured by BCA assays. The protein samples and ladder were run on 3–8% Tris-acetate gels without reducing agent at 150 V for 1.5 hours. The proteins were transferred onto Immobilon-P PVDF membranes overnight at 4 °C. Following transfer, the membranes were blocked with 5% milk in 1X Tris-buffered saline with 0.1% Tween-20 (TBST) and immunoblotted for KCC2 (1:1000 rabbit polyclonal Ab49917, Abcam) and GAPDH (1:1000 rabbit polyclonal G9545, Sigma,). HRP-conjugated secondary antibodies were used before treatment with ECL substrate (ThermoFisher, 32106). Western blots were imaged using ImageQuant LAS 4000. Multiple images were taken with increasing exposure times to ensure a linear working range. Protein expression levels were measured using Fiji^37^ and normalized to GAPDH expression for analysis.

### Quantitative PCR and analysis

Progenitor cells were cultured with and without hanging inserts in 12-well plates for 7 and 21 DIV before RNA extraction using the RNeasy mini kit (Qiagen, 74106). Following cDNA synthesis from the RNA samples, qPCR reactions were set up using TaqMan gene expression assays for Tuj1 (Hs00801390_s1), KCC2 (Hs00221168_m1) and GABA A1 receptor subunit (Hs00971228_m1) using the CFX Connect™ Real-Time PCR Detection System (BioRad). Experiments were set up in triplicates. qPCR data from three experimental repeats were analyzed using REST 2009 (Qiagen) with Tuj1 as the reference gene. All values were normalized to the ‘no insert’ values for analysis.

### Statistical analysis

Statistical analyses for significance were performed using one-way ANOVA followed by *post-hoc* t-tests to determine significance between specific groups. P-values for t-tests have been reported throughout the text. For comparison, the Kruskal-Wallis test on the data comparing the percentage of cells responding to muscimol at 21 DIV under the different conditions found that these data were significant with p-value < 0.0001. Post-hoc Mann-Whitney tests found that (1) the percentage of cells responding to muscimol at 21 DIV in the absence of retinal suspension (no insert) was significantly lower than those in the presence of P5 retinal suspension (P5 insert); p < 0.0001; (2) the percentage of cells responding to muscimol at 21 DIV in the presence of P5 retinal suspension was significantly higher than those in the presence of P14 suspension (P14 insert); p < 0.0001; and (3) the percentage of cells responding at 21 DIV with no retinal suspension was not significantly different from those cultured in the presence of P14 retinal suspension (p = 0.6124).
